# Supplementary material for: Nitric oxide donor sodium nitroprusside serves as a source of iron supporting Pseudomonas aeruginosa growth and biofilm formation
Source: Microbiol Spectr. 2025 Sep 16;13(10):e02234-25. doi: 10.1128/spectrum.02234-25 (PMC12502759; doi:10.1128/spectrum.02234-25)
Supplement: Supplemental material — Supplemental methods and Fig. S1 to S3. [file spectrum.02234-25-s0001.pdf]

SUPPLEMENTARY MATERIAL:

Text S1 - **Methods**

1.1 Bacterial strains and culture conditions

Pseudomonas aeruginosa PAO1 WT (HER-1018 P1). Cultures were routinely grown overnight in LB (lysogeny broth) media at 37ºC, 200rpm from stock glycerol cultures stored at -80ºC. Fresh M9 media (9 mM NaCl, 22 mM KH_2PO_4, 48mM Na_2HPO_4, 19mM NH_4Cl and 2 mM $MgSO_4$, 100 µM $CaCl_2$, 0.4% glucose, pH 7.0) were prepared prior to each experiment.

1.2 NO-donor preparation

50mM Sodium nitroprusside (SNP, CAT#71778-100G, Sigma-Aldrich) solutions were prepared in water. Spermine-NONOate (SP-NONO, CAT#0634655-16 (Z)-1-[N-[3-aminopropyl]-N-[4-(3-aminopropylammonio)butyl]-amino]diazen-1-ium-1,2-diolate) (Cayman Chemical) was dissolved in 10mM NaOH and stored at -20ºC. Stock solutions were used within 3 months.

1.3 Growth curve assays

Overnight *P. aeruginosa* PAO1 cultures were diluted to a OD_{600} of 0.05 in M9 media, supplemented with 65.25 µM SNP or $FeSO_4$. 200µl were inoculated in the wells of 96-well plates (2 biological replicates, 8 technical replicates) under aeration, (37ºC, 180rpm) over 24h. Cell density was recorded every 15 minutes for 24h by measuring OD_{600} using a CLARIOStar Plus microplate reader (BMG LabTech). For iron-depleted growth assays, M9 media was prepared as described above and supplemented with the metal chelator 2,2'-bipyridyl (CAT#D216305-10G, Sigma-Aldrich) to a final concentration of 500 µM. At 24h, two wells of each growth condition were transferred

to a 96-well plate and serially diluted 10-fold in PBS. 5µl were spot plated in triplicate in LB agar and incubated overnight. CFU counts were averaged and used to calculate the respective cell density at 24h in each culture.

1.4 Biofilm assays

Initial 10^7 CFU/ml bacterial solutions were prepared from overnight cultures using M9 media alone or supplemented with SNP or $FeSO_4$ and 1ml was added to each well of 24-well microtiter plates (Nunc, ThermoFisher Scientific). Biofilms were cultured in a shaking incubator (37ºC, 180rpm) within a container to maintain humidity. For biofilm dispersal assays, biofilms were incubated for 4h as described above and SP-NONO was subsequently added to the desired final concentrations (200 – 3.15 µM) and incubated for 15 minutes. Matching volumes of a 10mM NaOH solution were added as a vehicle control. Following a washing step with 1ml of PBS, biofilms were stained with 0.1% crystal violet dissolved in 6.25% methanol. Stained biofilms were washed twice with PBS and the remaining dye was solubilised with absolute ethanol. Biofilm biomass was quantified via OD_{550} readings in a SPECTROStar Nano microplate reader (BMG LabTech). Additional biofilm biomass measurements accounting for surface-attached cells were measured by washing each well as previously described and then adding 1ml of fresh PBS to each well prior to sonicating each plate for 20 minutes on ice-cold water. Bacterial suspensions in each well were then serially diluted 1:10 in PBS and plated in triplicate on LB agar for CFU counting. Each assay was performed at least with 2 biological repeats and 2 technical repeats.

1.5 Statistical analyses

Statistical analyses were performed by One-Way ANOVA followed by a Dunnett's post hoc test using Graphpad Prism10 (Graph Pad, La Jolla, CA, USA).

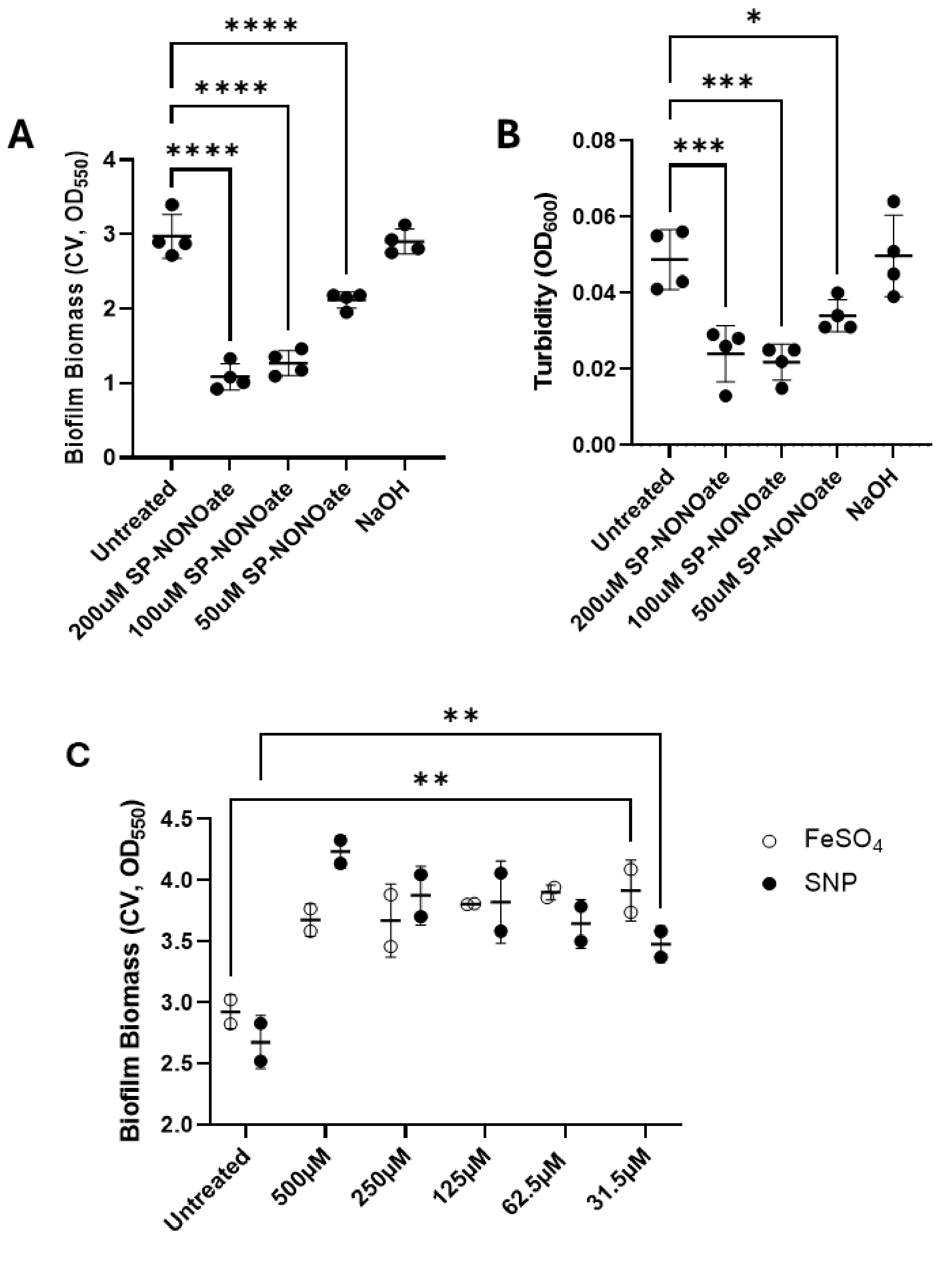

Fig S1. SP-NONOate induces a dose-dependent *Pseudomonas aeruginosa* biofilm dispersal response whereas SNP and iron increase biofilm biomass. 4h-old biofilms of *P. aeruginosa* PAO1 were grown in M9 media using 24-well microtiter plates. Ranging concentrations of SP-NONOate were added for 15-min **(A, B)**, or $FeSO_4$ or SNP **(C)** for 30-

min. Biofilm biomass was measured by crystal violet staining **(A, C)** and attached CFU by OD600 **(B).** Asterisks indicate a statistically significant difference between treated and untreated groups of the same treatment type (*, $P<0.05$, ***, $P<0.001$; ****, $P<0.0001$), calculated by Ordinary One Way ANOVA with a Dunnett's Multiple Comparisons test. At least two independent cultures were included. Biofilm biomass was recorded by crystal violet staining after treatment times were completed. The means ± SD are represented in the graph.

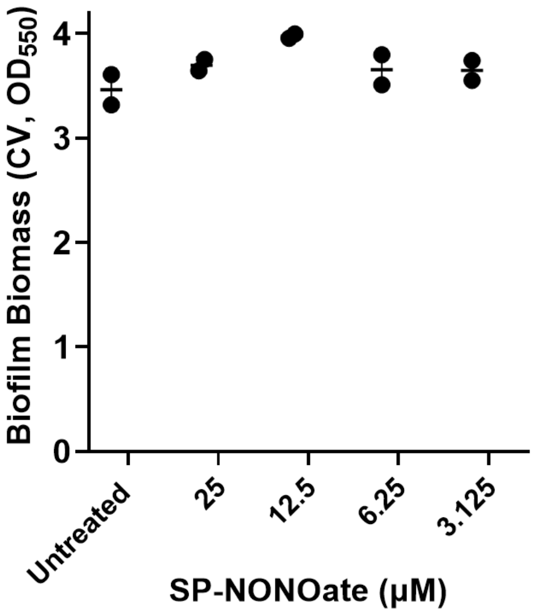

Fig S2. Sub-dispersing concentrations of NO by SP-NONOate do not elicit biofilm biomass accumulation. 4h-old biofilms were grown in M9 media using 24-well microtiter plates. After 4h of incubation, ranging concentrations of SP-NONOate were added to the desired final concentrations for 30 min and biofilm biomass was recorded by crystal violet staining. Statistical differences were calculated by Two Way ANOVA with a Dunnett's Multiple Comparisons test. Two independent cultures were included. The means ± SD are represented in the graph.

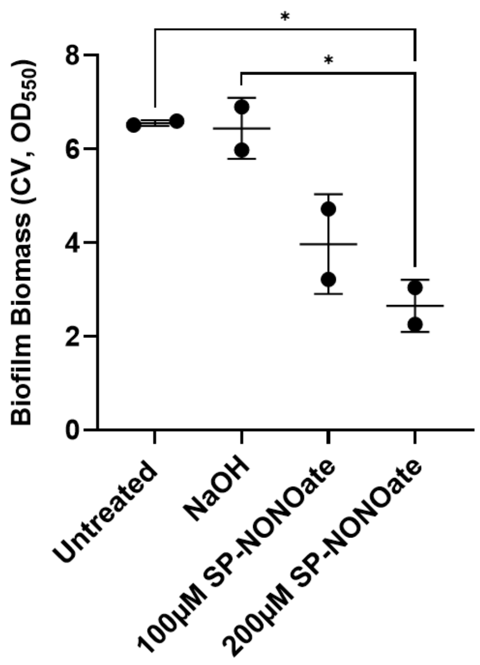

Fig S3. NO inhibits biofilm formation of *P. aeruginosa* PAO1 biofilms. 4h-old biofilms were grown in M9 media using 24-well microtiter plates with SP-NONOate. Biofilm biomass was recorded by crystal violet staining. Statistical differences were calculated by Ordinary One Way ANOVA with a Dunnett's Multiple Comparisons test. Two independent cultures were included. The means ± SD are represented in the graph.
